# Case report: Identification of atypical mantle cell lymphoma with *CCND3* rearrangement by next-generation sequencing

**DOI:** 10.3389/fonc.2023.1145376

**Published:** 2023-03-29

**Authors:** Luomengjia Dai, Han Zhang, Wen Chen, Yi Xia, Shuchao Qin, Yang Shao, Jianyong Li, Yi Miao, Bingzong Li, Huayuan Zhu

**Affiliations:** ^1^ Department of Hematology, the First Affiliated Hospital of Nanjing Medical University, Jiangsu Province Hospital, Nanjing, China; ^2^ Pukou Chronic Lymphocytic Leukemia (CLL) Center, Pukou division of Jiangsu Province Hospital, Nanjing, China; ^3^ Jiangsu Key Lab of Cancer Biomarkers, Prevention and Treatment, Collaborative Innovation Center for Cancer Personalized Medicine, Nanjing Medical University, Nanjing, China; ^4^ Department of Hematology, the Second Affiliated Hospital of Soochow University, Suzhou, China; ^5^ Department of Pathology, the First Affiliated Hospital of Nanjing Medical University, Jiangsu Province Hospital, Nanjing, China; ^6^ Geneseeq Research Institute, Nanjing Geneseeq Technology Inc., Nanjing, China

**Keywords:** mantle cell lymphoma, CCND3 rearrangement, NGS, diagnosis, fusion gene

## Abstract

The t(11;14) (q13;q32) translocation resulting in overexpression of cyclin D1 is the major oncogenic mechanism in mantle cell lymphoma (MCL). Most MCLs can be diagnosed based on morphological features, cyclin D1 expression, and IGH/CCND1 rearrangement. However, in some atypical cases where conventional FISH studies fail to detect IGH/CCND1 rearrangement or immunohistochemistry for cyclin D1 is negative, the diagnosis of the disease can be difficult. Hence, next-generation sequencing (NGS) may allow the identification of molecular alterations and assist in the diagnosis of atypical MCL. In this study, we reported a case of a patient diagnosed as asymptomatic MCL who presented with lymphadenopathy during the initial assessment. A lymph node biopsy was performed and the results revealed a high Ki67 index. However, initial diagnosis of aggressive MCL was difficult since the IGH/CCND1 rearrangement result was negative. Ultimately, by the aid of NGS we identified a rare *CCND3* rearrangement in the patient, which lead to overexpression of cyclin D3, thereby facilitating the diagnosis of MCL.

## Introduction

Mantle cell lymphoma (MCL) is a type of mature B-cell lymphoma with variant clinical presentation and molecular features. Morphologically, classical MCL consists of small to intermediate-sized cells with irregular and cleaved nuclei, dense chromatin and indistinct nucleoli. Immunophenotypically, MCL cells typically express CD5, CD20, CD19, sIgM/sIgD, FMC-7, SOX11 and cyclin D1 with monoclonal kappa/lambda light chains while CD23 and CD200 are usually negative. Acquisition of genetic alterations may promote cell survival and proliferation, which lead to more aggressive forms of MCL (1). These aggressive variants are morphologically characterized by neoplastic cells resembling lymphoblasts with a high Ki67 index in affected sites (2).

Most MCLs are characterized by the t(11;14) (q13;q32) translocation, which results in overexpression of cyclin D1. Nevertheless, several cyclin D1-negative cases have been identified in the past (3–6). FISH analysis can reveal t(11;14) (q13;q32) translocation with IGH/CCND1 fusion probe. However, cryptic *CCND1* re-arrangement with light chain partners or translocations involving *CCND2* and *CCND3*, which alternatively contribute to pathogenesis of MCL presented with negative results of IGH/CCND1 rearrangement. In these cases, break-apart probes for *CCND1*, *CCND2*, *CCND3*, *IGH*, *IGK*, and *IGL* can be used. Immunostains for cyclin D2, cyclin D3 or cyclin E may reveal overexpression of certain cell cycle regulatory proteins. Besides, gene expression profiling can be used to refine the diagnosis of MCL. Characteristic MCL gene expression signature can be detected by cDNA microarray expression analysis or quantitative reverse transcriptase-polymerase chain reaction (RT-PCR) in cyclin D1-negative lymphoma, thus supporting the diagnosis of cyclin D1-negative MCL (3). Similarly, NGS can also be useful in detecting rare or cryptic rearrangements, thereby facilitating the diagnosis of MCL.

In this study, we reported a cyclin D1-negative MCL patient and discussed how the diagnosis was finally established. This study was approved by the Institutional Review Board of the First Affiliated Hospital of Nanjing Medical University Ethics Committee and a written informed consent was obtained from the patient.

## Case presentation

**Figure 1:**
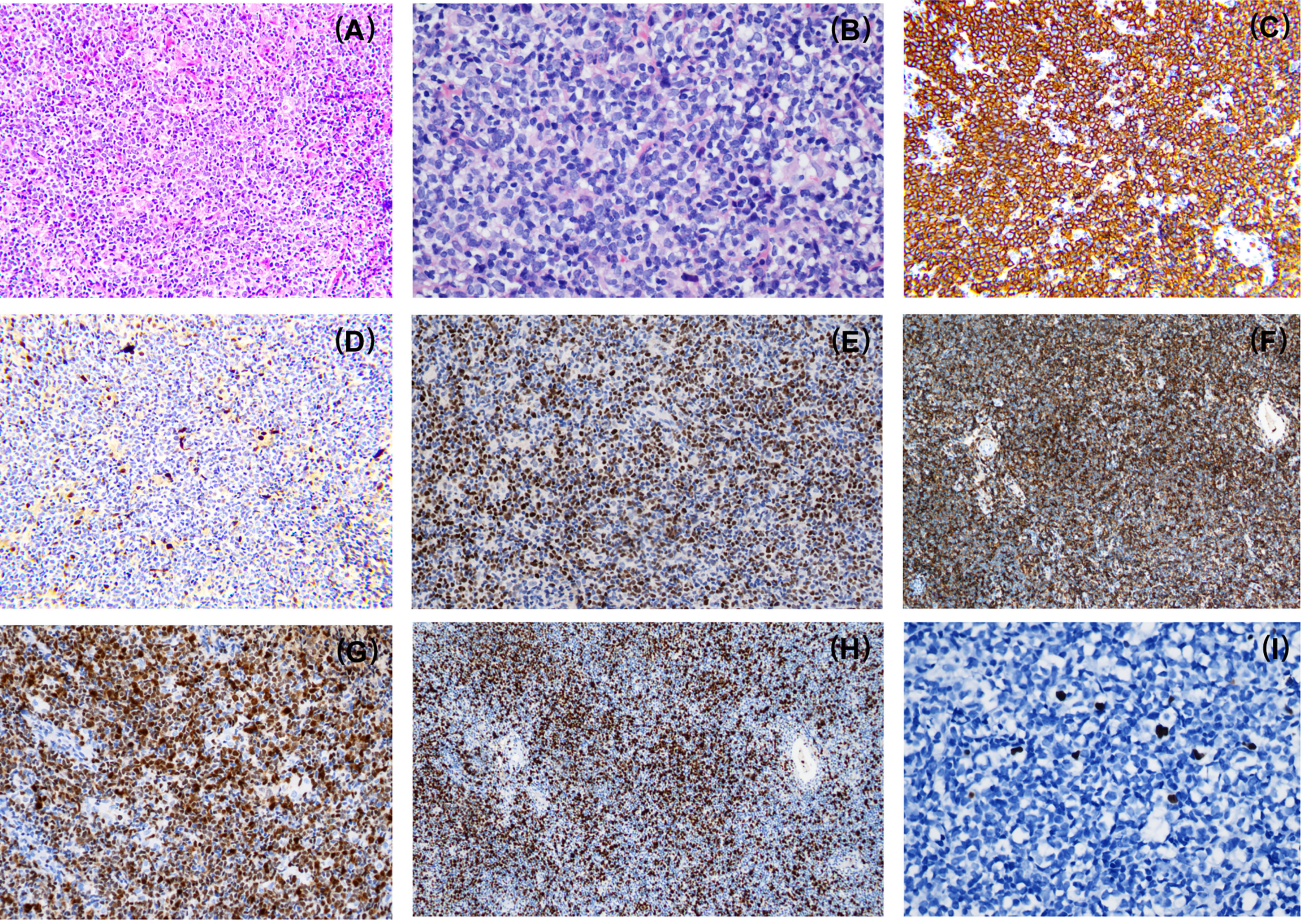
Morphologic and immunophenotypic evaluation of case1. Pathological examination showed architectural effacement by medium-sized tumor cells **(A)**. The lymphoma cells have a pleomorphic morphology **(B)** and express CD20 **(C)**, SOX11 **(E)**, CD43 **(F)**, MUM1 **(G)** and a high Ki67 proliferation index **(H)** while the lymphoma cells are negative for cyclin D1 expression **(D)**. EBV-encoded RNA (EBER) was negative **(I)**. A,E,F,G,H×100; C,D×200; B,I×400.

**Figure 2:**
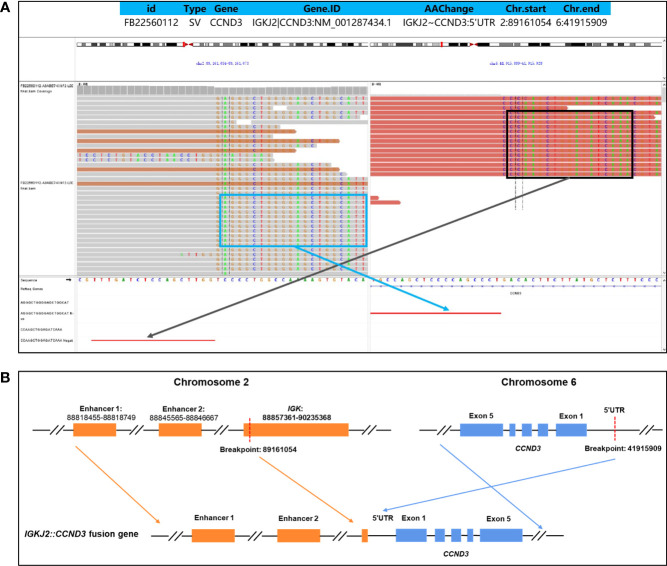
Identification of an IGKJ2::CCND3 fusion by DNA-targeted NGS. The formalin-fixed paraffin-embedded (FFPE) tissue in primary tumor tissues and plasma cell-free DNA (cfDNA) of pretreatment were detected by targeted DNA sequencing of 475 leukemia- and lymphoma-related genes. The Delly fusion calling tool was used to identify the number of chimeric reads (sequencing paired ends mapped to different genes) and split reads (spanning a fusion breakpoint) from the targeted DNA-seq data, and each SV was manually checked on the Integrative Genomics Viewer (IGV). The structure variations (SVs) profile showed a rearrangement between chromosome 2 and chromosome 6. The exact fusion breakpoints are localized at chr2: p11.2: 89,161,054 and chr6: p21.1: 41,915,909, respectively. Sequences supportive of IGKJ2::CCND3 fusion were detected by targeted DNA sequencing with a mutant allele frequency of 30.1% in tissue and 12.5% in plasma cfDNA **(A)**. Schematic genome rearrangement of IGKJ2::CCND3 revealed that the exon 1-exon 5 remained completely intact, and two enhancers of chromosome 2 located upstream of CCND3, which may lead to a high expression of CCND3 protein **(B)**.

The patient was a 43-year-old male who presented with lymphadenopathy at our hospital. Laboratory tests indicated lymphocytosis and elevated serum lactate dehydrogenase (LDH). A positron emission tomography (PET)/computed tomography (CT) scan showed generalized lymphadenopathy in the head, neck, axilla, mediastinum, abdomen, and groin. A large mass (approximately 15*7.5cm) was seen in the abdominal cavity and the maximum standardized uptake value (SUVmax) of the mass was 9.0. Segmental elevated FDG uptake suggested involvement of the gastrointestinal (GI) tract. A core biopsy of the lymph node was performed and pathological examination showed architectural effacement by medium-sized tumor cells, which showed scant cytoplasm, oval irregular nuclei, fine chromatin, and inconspicuous nucleoli. Flow cytometry immunophenotyping indicated that the tumor cells were positive for CD5, CD19, CD20, FMC7, and restricted lambda light chain expression and negative for CD23 and CD200. By immunohistochemistry (IHC),the tumor cells were positive for CD5, CD20, CD43, BCL6, MUM1,BCL2, MYC, and SOX11 and negative for cyclin D1, CD10, EBER, and LEF-1 (**Figure 1**). The ki67 index was 70% (**Figure 1H**). Further, no IGH/CCND1 rearrangement was detected by using fluorescence *in situ* hybridization (FISH) analysis. Bone marrow smear showed a 14% involvement by lymphoblasts and pro-lymphocytes and additionally showed plasmacytoid lymphocytes, plasma cell infiltration and rouleaux red blood cells. The peripheral blood smear result was consistent with that of the bone marrow smear. IHC studies done on bone marrow samples showed that the tumor cells were positive for CD5 and CD20 and negative for cyclin D1, SOX11, CD10 and LEF1. The flow cytometric analysis of the bone marrow was consistent with that of the lymph node. Chromosomal karyotype analysis of bone marrow samples showed a normal karyotype (46, XY [15]). IGHV status was unmutated. The diagnosis of pleomorphic MCL was suspected. NGS was performed using plasma, bone marrow and lymph node tissue samples. IGKJ2~CCND3 gene fusion was detectable in all samples (**Figures 2A, B**). RNA-seq on lymph node tissue reconfirmed this fusion. The final diagnosis of MCL was established. The patient was treated with zanubrutinib, lenalidomide and rituximab plus cyclophosphamide, doxorubicin, vincristine, and prednisone (ZR^2^-CHOP) and achieved a complete clinical and molecular response after six cycles, and followed by rituximab maintenance every two months. With a follow-up of 8-9 months, the patient is still in remission.

## Discussion

In this study, we identified an atypical MCL patient with a CCND3 rearrangement by NGS. The expression of CD5 and SOX11 points to the diagnosis of MCL. As a specific diagnostic marker, SOX11 is frequently expressed in MCL but is absent in DLBCL, thereby distinguishing MCL from DLBCL. Additionally, SOX11 expression is also helpful for identifying cyclin D1-negative MCL (7). Although cyclin D1 expression was negative in this particular case, the presence of IGKJ2/CCND3 gene fusion further confirmed the diagnosis of MCL. In addition to the above evidence, other clues also supported the diagnosis of MCL. For example, CD43 is expressed in more than 90% of MCL cases but in only 25% of DLBCL cases (8, 9). Therefore, the CD43 expression in this case supported the diagnosis of MCL. Further, the unmutated IGHV status also made the diagnosis of DLBCL less likely.

As a matter of fact, diagnosis of MCL is well established. In most cases, experienced hematopathologists can easily establish the diagnosis based on distinct morphologic features and immunophenotyping. The molecular hallmark, IGH/CCND1 rearrangement is detectable in 90~95% of MCL cases and results in overexpression of cyclin D1 (10). This abnormality can be detected by FISH using dual-color dual-fusion probes. Although in rare cases, cryptic rearrangements with light chain partners do exist and this can be detected by CCND1 break-apart probes (11, 12). In such a situation, the overexpression of cyclin D1 by IHC may help to establish a diagnosis. In the majority of cases, the characteristic FISH results and cyclin D1 expression are helpful in the diagnosis of MCL. However, in cases without either of the positive findings, the diagnosis of MCL could be challenging. It is reported that *CCND2* or *CCND3* rearrangement occurs in 93% of cyclin D1-negative MCL (6). *CCND2* rearrangements are the most frequent genetic events in cyclin D1-negative MCL (13) while *CCND3* rearrangements are relatively rare. *CCND2* or *CCND3* rearrangements result in overexpression of cyclin D2 and cyclin D3 respectively, both of which may in turn alternatively serve as the initial hit event in MCL pathogenesis. In actual fact, the clinical presentation and gene expression profiling of these cyclin D1-negative cases are like those of cyclin D1-positive MCL (3, 10). Break-apart probes for *CCND2*, *CCND3*, *IGH*, *IGK*, and *IGL* can be used in these cases and gene expression profiling may further support the diagnosis.

NGS could assist in the diagnosis of MCL especially when the FISH analysis result is negative. Petersons et al. once reported a case of MCL with a cryptic *CCND1* rearrangement that is not detected by FISH due to the minimal size of the inserted gene segment (14). NGS instead identified the small insertion of *CCND1* into the IGH locus, thereby confirming the presence of IGH/CCND1 rearrangement. Similarly, cryptic *CCND3* rearrangements (negative for FISH with *CCND3* break-apart probe) have been identified in 3 cyclin D1-negative cases and NGS verified IGK/L enhancers hijacking in these cases (6). In addition to the detection of translocations, NGS is capable of detecting rare mutations, inversions, insertions, deletions, copy number variants and etc., thereby providing a comprehensive picture of molecular aberrations.

In conclusion, traditional morphological and immunophenotypic strategies combined with cytogenetic characteristics and molecular profiling are required for the accurate diagnosis of atypical MCL. For cases highly suspected of MCLs, NGS method can be further utilized to detect rare rearrangements and assist the diagnosis.

## Data availability statement

The datasets presented in this article are not readily available because of ethical/privacy restrictions. Requests to access the datasets should be directed to the corresponding author.

## Ethics statement

The studies involving human participants were reviewed and approved by the First Affiliated Hospital of Nanjing Medical University Ethic Committee. The patients/participants provided their written informed consent to participate in this study. Written informed consent was obtained from the individual(s) for the publication of any potentially identifiable images or data included in this article.

## Author contributions

HuZ, YM, BL conceived the idea for the article. LD and HaZ drafted the original manuscript. YM and HuZ approved the final version of the manuscript. WC and YS performed pathologic and molecular diagnostic testing. All authors participated in patient management and data collection. All authors contributed to the article and approved the submitted version.
